# Gamified Mobile App (MobERAS) for Telemonitoring Patients in the Postoperative Period Based on the Enhanced Recovery after Surgery Program: Development and Validation Study

**DOI:** 10.2196/56033

**Published:** 2024-08-14

**Authors:** Aline Evangelista Santiago, Victor Pezzi Gazzinelli Cruz, Rafaela Souza Furtado, Eduardo Batista Cândido, Wladmir Cardoso Brandão, Agnaldo Lopes Silva Filho

**Affiliations:** 1 Department of Gynecology, Obstetrics and Mastology Faculdade de Medicina de Botucatu Universidade Estadual Paulista “Júlio de Mesquita Filho” Botucatu Brazil; 2 Department of Computer Science Pontifícia Universidade Católica de Minas Gerais Belo Horizonte Brazil; 3 Department of Gynecology and Obstetrics Universidade Federal de Minas Gerais Belo Horizonte Brazil

**Keywords:** handheld computer, mobile phone, postoperative period, mHealth, mobile health, telemedicine, postoperative, perioperative, recovery, surgery, surgical, gamify, gamified, gamification, app, apps, application, applications, design, develop, development, gynecology, gynecological, oncology, oncological, women’s health, usability

## Abstract

**Background:**

Digital technology and gamified apps can be useful in the health care context. Gamification uses technology to influence users’ actions and motivations through experiences that resemble games. Patient adherence to the enhanced recovery after surgery (ERAS) program is crucial for achieving early recovery after surgery and continuous monitoring is essential for obtaining good results.

**Objective:**

This study aimed to describe the development and validation of a mobile app for enhanced recovery after surgery (MobERAS), a gamified mobile health app for telemonitoring patients in the postoperative period based on the ERAS program, and to evaluate its functionality and usability and the experience of patients, health care professionals, and computer professionals with its use.

**Methods:**

We developed MobERAS for postoperative telemonitoring, with active participation of patients in the process, and offering availability of real-time information for the health team. The app development process included idealization, interdisciplinary team formation, potential needs assessment, and product deployment. Usability tests were conducted throughout the development process with improvements, technical adjustments, and updates. After finalization, comprehensive verification tests were performed. The parameters evaluated are those that can influence the length of hospital stay, such as nausea, vomiting, pain scales, return to normal gastrointestinal function, and thromboembolic events. MobERAS was designed to be downloaded by users on their phones, tablets, or other mobile devices and to provide postoperative data. The app has a GPS that monitors the patient’s walking time and distance and is connected to a virtual database that stores the collected data.

**Results:**

Women undergoing medium and major gynecologic oncologic surgeries were included. We included 65 patients with an average age of 53.2 (SD 7.4, range 18-85) years. The time of use ranged from 23.4 to 70 hours (mean 45.1, SD 19.2 hours). Regarding adherence to the use of MobERAS, the mean fill rate was 56.3% (SD 12.1%, range 41.7%-100%), and ambulation data were obtained for 60 (92.3%) of the 65 patients. The researcher had access to the data filled out by the patients in real time. There was good acceptance of the use of MobERAS by the patients, with good evaluation of the app’s usability. MobERAS was easy to use and considered attractive because of its gamified design. The app was rated as good or very good in all items by health care professionals (n=20) and professionals specializing in technological innovation (n=10).

**Conclusions:**

MobERAS is easy to use, safe, well accepted by patients, and well evaluated by experts. It can be of great use in clinical surgical practice and an important tool for greater engagement of patients and health care professionals with the ERAS program.

## Introduction

The usefulness of digital technology in the health field has been well recognized for its great potential to aid in the prevention, diagnosis, and management of diseases [1,2]. Moreover, given that the COVID-19 pandemic has promoted digital disruption worldwide, further incorporation of technology into health care practices can be expected [3].

Through mobile apps, patients can access health-related data, schedule medical appointments, manage medication dosages, improve well-being, and perform other health-related activities [1]. Many of these apps are aimed at patients with chronic illnesses, such as diabetes mellitus, obesity, mental disorders, malignant neoplasms, smoking, and alcoholism, leading to apparent improvements in self-management of the disease and aiding in promoting health [4-11]. Therefore, the use of technology to improve health is promising [12].

A *mobile medical app* incorporates the functionality of the device’s software or transforms a mobile platform into a regulated medical device [13]. In this scenario, there is gamification, which consists of using technology to influence users’ actions and motivations through experiences that resemble games [14]. Gamified apps have several purposes in the context of health care, including managing comorbidities, preventing disease, encouraging the practice of healthy lifestyle habits, providing information, and building character [12]. Associating the gamification proposal to stimulate some practices and the need for better adoption of medical recommendations, we proposed the development of a gamified medical app, which represents 1 strategy for adopting the measures recommended by the enhanced recovery after surgery (ERAS) program and for promoting greater patient engagement.

The ERAS program comprises multimodal perioperative assistance designed to achieve early recovery of patients undergoing surgery, with the objective of reducing hospitalization time and accelerating the return of patients to regular activities without increasing complications, hospital readmission rates, or costs. For better recovery, the program focuses mainly on reducing perioperative stress, satisfactory pain control, return to normal gastrointestinal function, and early mobilization. Adherence to the program is crucial, and continuous monitoring is essential for obtaining good results. Among the program’s postoperative recommendations, patients are encouraged to follow some steps that can help them achieve good postoperative results, such as encouraging early mobilization, as well as early oral intake [15,16]. For example, it is known that the incentive to walk early exerts a positive impact on the incidence of thromboembolic events [17].

Aiming at optimized postoperative recovery and using the current concept of mobile technology in health, we developed an app (mobile app for enhanced recovery after surgery [MobERAS]) for postoperative telemonitoring, with active participation of the patients in this process and the availability of real-time information for the health team. MobERAS is used as a tool to simultaneously guide and encourage patients to follow medical recommendations through the use of gamification. This study aimed to describe the development and validation of MobERAS, evaluating its functionality and usability and the experience of patients, health care professionals, and computer professionals with its use.

## Methods

### Development Process

#### Development Approach and Regulatory Context

Determining the most appropriate development strategy was challenging, given the existence of different sources of guidance and few well-established regulations. To establish the app’s development strategies, we followed the recommendations of the Regulation of the European Parliament, which set high quality and safety standards for medical devices. According to the Regulation of the European Parliament, MobERAS can be defined as a “medical device, because it is a software designed for specific medical purposes, such as diagnosis, prevention, and monitoring, without the use of pharmacological, immunological, or metabolic means.” The device must be safety and efficacious, without compromising the clinical condition of patients. Risks must be managed such that they are minimized and considered acceptable. According to the European Regulation classification rules, MobERAS belongs to the class IIa category or moderate risk, as it is a noninvasive app designed to provide information for decision-making, diagnostic purposes, or therapeutic purposes without causing any immediate danger to the patient [18].

Under the specific definitions, MobERAS can be classified as an “active device intended for diagnosis and monitoring,” as it is an active device for monitoring physiological processes and health conditions in the postoperative period. To meet the regulatory requirements of the class IIa category, general safety and performance adjustments are required, in addition to delivery of clinical evidence. To deal with general security and performance requirements, it is necessary to describe the software design and validation process with information that allows the project stages to be understood [18].

To comply with these regulations, a MobERAS validation study was performed. The app development process included (1) idealization; (2) interdisciplinary team formation; (3) assessments of potential needs, feasibility, user population, and concept finalization; (4) app construction and product development; and (5) validation.

Apps, in general, must be usable in different countries; therefore, cultural and linguistic differences must be considered during their development [19]. Thus, MobERAS was developed in Portuguese for validation in Brazil using internationally validated scales, such as the visual analog scale for pain, as well as figures representative of the respective health status, enabling translation into other languages and interpretation by different cultures. A new version in English and Spanish has already been planned.

#### Concept Development and the Use of Gamification

The concept of MobERAS emerged from the need to encourage patients to comply with medical recommendations in the postoperative period. According to the ERAS program, active participation of patients in their recovery process is essential for obtaining good outcomes [16]. Accordingly, the app was designed such that patients could receive guidance on recommendations to follow in the postoperative period and be monitored and encouraged to adopt the targeted measures. In this context, the development of a gamified app was initiated.

By combining gameplay mechanics and experiences, gamification creates situations that resemble games, such as reward systems, using points, achievement badges, and leaderboards. The idea was to increase participation and promote the engagement and commitment of patients in their postoperative care through gamification. Encouraging measures such as early ambulation and oral intake improves the return to normal gastrointestinal function, decreases thromboembolic event risks, promotes early hospital discharge, and reduces complication rates [16]. To improve patient adherence to medical recommendations, the artifice of gamification was added. For example, when a patient positively fills in an item or walks for more time (>5 minutes) or a longer distance (>10 steps), they earn a bonus represented by stars or an incentive animation, similar to that in a game. Accordingly, goals are established with a reward-based strategy to obtain results, encouraging the patient to comply with medical recommendations.

#### Interdisciplinary Research Team

Interdisciplinarity in research involves sharing scientific knowledge among research team members [19]. In this study, intercommunication of the health and information technology areas was important. Researchers with training in information technology and those with experience in postoperative care were involved. The research team included a computer science professor, a computer science graduate student, a medical gynecologist oncology professor, a doctoral student in oncological gynecology, and a medical student.

All the researchers studied the ERAS program. After several meetings by the professionals involved in the project, the app’s focus was reached. Goals to be achieved by patients were also defined. Subsequently, the app’s functions, interface, and design were determined.

#### Assessments of Potential Needs, Feasibility, User Population, and Concept Finalization

According to ERAS program recommendations, the parameters evaluated in postoperative monitoring—simple interventions, such as early feeding, early ambulation, and multimodal analgesia—have shown to decrease the rate of postoperative ileus [20]. In the 1990s, Kehlet [21] introduced the idea of a multimodal approach to enhance functional rehabilitation postoperatively. One study suggested that avoiding limiting procedures (eg, using a urinary catheter or excessive venous hydration), implementing practices involving the use of local analgesia, and encouraging early nutrition can lead to faster postoperative recovery, as well as reduced morbidity and costs [22]. The recommendations are summarized in Table 1.

**Table 1:** Recommendations of the ERAS^a^ program considered during app conceptualization.

Item	ERAS recommendations
Venous catheter	Remove venous catheter when patient tolerates 500 cc of oral diet
Diet	Regular diet in immediate postoperative period Oral hydration
Ambulation	Walk 8 times a day Have all meals sitting in a chair Stay out of bed for 8 hours a day
Bladder catheter	Remove on first postoperative day
Nausea and vomiting/pain	Multimodal approach Avoid opioids

^a^ERAS: enhanced recovery after surgery.

**Figure 1:**
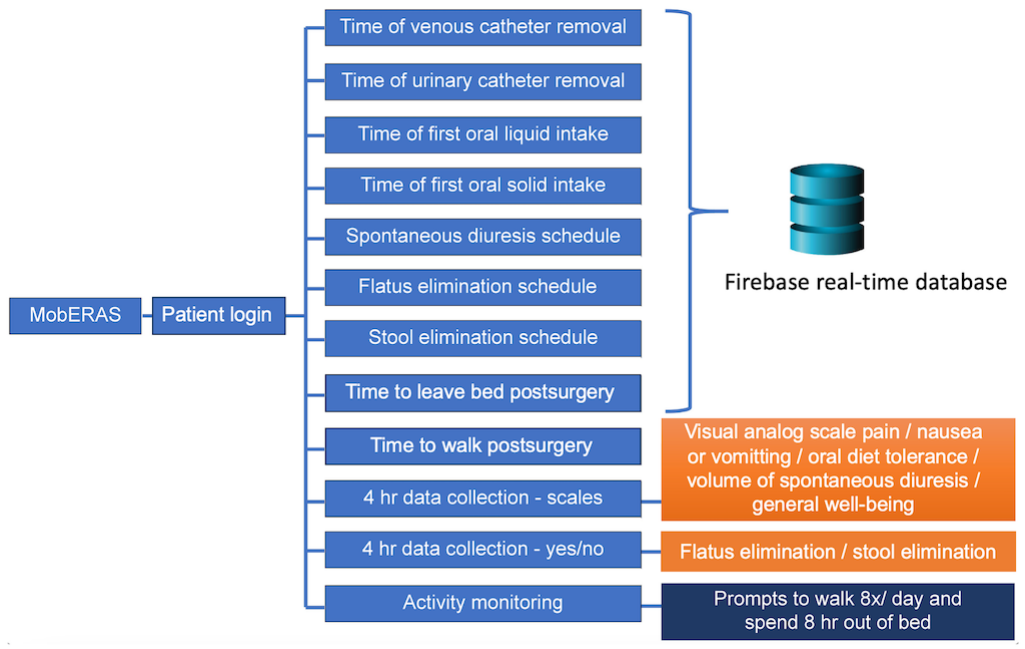
Summary of postoperative parameters assessed by MobERAS created for this study based on the ERAS protocol. ERAS: enhanced recovery after surgery; MobERAS: mobile app for enhanced recovery after surgery.

Thus, these parameters were defined as those to be assessed in the first version of the product (Figure 1). Through frequent and intermittent alerts triggered by the app, patients are encouraged to follow medical instructions, such as walking and moving their legs, and to complete assessments in the form of scales evaluating pain, nausea, and vomiting. Moreover, the app has a GPS that monitors the patient’s walking time and distance.

**Figure 2:**
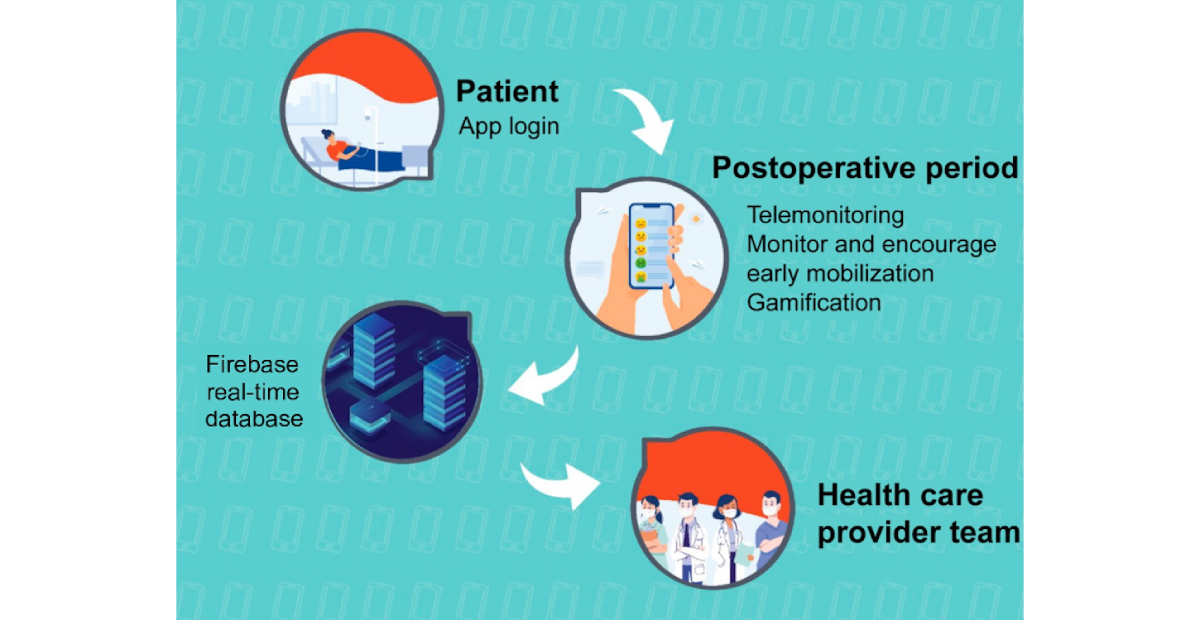
MobERAS enables real-time telemonitoring of the postoperative course and aims to improve patients' adherence to medical recommendations using gamification tools. MobERAS: mobile app for enhanced recovery after surgery.

The ERAS program and, therefore, MobERAS are applicable to patients undergoing elective surgeries in general, and MobERAS was developed for use in the postoperative period. Patients can download the app on their phones, tablets, or other mobile devices. There is an easy-to-understand login that requests the user’s name, individual registration (in Brazil, the “Cadastro de Pessoas Físicas”), and date of birth. The information provided by the patient can be accessed remotely in real time by the assistant health care team through cell phones (Figure 2). Thus, the app functions as a health care tool for patients.

After a detailed elaboration of the product concept, an app development plan was generated, including issues such as design, usability, validation, and integration with the online database, in addition to assessment of possible risks. The product concept and development plan included functional and nonfunctional design requirements based on stakeholder specifications, such as architecture documentation, software integration, and unit test specifications.

For attractiveness, mobile device technology needs to be easy to navigate, be self-explanatory, and not contain much screen text [23]. Moreover, user instructions must be written for easy understanding and, when appropriate, supplemented with figures and diagrams [18]. Thus, an app was designed with multiple playful resources, such as symbols and images, with the minimal use of words. The texts were written using a language as close as possible to the popular colloquial language, without the use of many technical terms that could make it difficult for patients to understand. Furthermore, there was significant focus on developing an app that was easy to navigate, as some target patients were elderly and possibly unfamiliar with advanced and complex technologies.

As the app was designed with several playful resources, it can be translated and applicable in other countries without the need for additional programming. The app was built using the backend system and the Flutter framework. The app is connected to a virtual database (Firestore) to store the collected data. Every answer provided by the patient and all walking times are stored in this database on the internet for further analysis. The text used by the graphical user interface is managed separately to ensure easy software upgrades (eg, providing the ability to add new languages by simply translating the source text without additional programming).

The source code of the MobERAS system is available at Ref. [24].

### Validation Process

To validate MobERAS, the study included the participation of a multidisciplinary team, including physicians and nurses from the health care team, computer professionals, and patients who used the app.

#### Study Population

Although the app is applicable to several surgical specialties, it was validated in the patient population attended by the physicians participating in the study. Patients with proposed medium and major gynecologic oncologic surgeries were invited to participate in the study. The patients were recruited from a public teaching hospital and a private hospital, both in Brazil and references in the care of women with gynecological cancer.

In total, 69 patients were invited to participate in the study, all of whom were undergoing medium and major gynecologic oncologic surgery. Of these, 4 (5.8%) patients refused to participate in the study: 2 (50%) of these 4 patients argued that they had no contact with any mobile device. These were an 83-year-old and a 72-year-old patient, the first one with no educational background and family income of 1 minimum wage and the second with incomplete primary education and family income between 1 and 2 minimum wages. Thus, 65 (94.2%) patients were included in the study, 35 (53.8%) patients from the public teaching hospital and the other 30 (46.2%) from the private hospital.

#### MobERAS Functioning

MobERAS was tested in the postoperative period as soon as patients arrived in the postanesthetic recovery room. The time of use was determined by the length of hospitalization of patients in the postoperative period. We evaluated adherence to the use of the app through the percentage of completion of the requested topics and through data collection regarding mobility and ambulation.

#### Patients’ Evaluation of MobERAS

Aiming at evaluating the usability of MobERAS and its acceptance by users, at the end of its use by patients, at the time of hospital discharge, the patients were invited to fill out a survey, evaluating their experience with using the app and providing their opinion about it (Table 2). For this evaluation, we used the System Usability Scale (SUS), a usability scale developed by Brooke [25] in 1986. The SUS is a simple scale that offers a global view of subjective evaluations of usability [25].

The SUS is composed of 10 statements that are scored on a 5-point agreement strength scale, where 1 means “I totally disagree” and 5 means “I totally agree.” The final scores range from 0 to 100, where higher scores indicate better usability. The statements alternate between positive and negative [25] because there are features in the statements that tend to result in generally positive or negative evaluations [26].

The SUS produces a single number (SUS score) that represents a measure of the overall usability of the system. Individual item scores are not meaningful in isolation. To calculate the SUS score, 1 is subtracted from the score that the user provides to the odd items (1, 3, 5, 7, and 9) and the user’s score provided to the even items (2, 4, 6, 8, and 10) is subtracted from 5. For example, if the user scores an even item 2, the item score is 5 – 2 = 3. Thus, the contribution score for each item ranges from 0 to 4. After this, all the scores of the 10 questions are added, and the total value is multiplied by 2.5, resulting in the final score [25]. A score above 68 is considered above average, and a value below 68 is below average. However, Despite the wide use of the SUS, there is little guidance on the interpretation of SUS scores [27].

**Table 2:** MobERAS^a^ evaluation questionnaire answered by the patients^b^.

Item #	Item	I totally disagree	I disagree	I neither agree nor disagree (indifferent)	I agree	I totally agree
1	I would use MobERAS again.					
2	I found MobERAS unnecessarily complex.					
3	I found MobERAS easy to use.					
4	I would need help from a person with technical knowledge to use MobERAS.					
5	I think MobERAS functions are very well integrated.					
6	I think MobERAS is too inconsistent.					
7	I imagine that people will learn how to use MobERAS quickly.					
8	I found MobERAS confusing to use.					
9	I felt confident using MobERAS.					
10	I had to learn several new things before I could use MobERAS.					

^a^MobERAS: mobile app for enhanced recovery after surgery.

^b^Adapted from Brooke [25].

#### Evaluation of MobERAS by Health Care and Computer Professionals

To evaluate the quality of the information contained and provided by MobERAS, we used a multidimensional scale developed by Stoyanov et al [28] to rate and evaluate the quality of mobile health (mHealth) apps [28]. According to the Mobile App Rating Scale (MARS), the app quality was evaluated in 4 dimensions (engagement, functionality, aesthetics/design, and information) by 20 health care professionals (physicians and nurses) and 10 professionals with a computer science background working in technological innovation. All items were evaluated on a 5-point scale, from 1 for “inadequate” to 5 for “excellent.” The minimum-possible score for each item was 30 and the maximum was 150 (1 and 5 points for each professional, respectively).

### Ethical Considerations

The study was approved by the Ethics Committee of both hospitals (Women's Hospital Prof. Dr. José Aristodemo Pinotti and Vera Cruz Hospital), in addition to the ethics committee of São Paulo State University “Júlio de Mesquita Filho” (approval number 98361118.0.0000.5411), the proponent institution of the research, and all participants signed an informed consent form.

## Results

### Constructing a Mobile App: Product Development

#### The Initial Product (Prototype)

An initial product (prototype) was developed to run on the Android operating system, based on Figures 1 and 2. Usability tests were performed throughout the development process, with improvements, technical adjustments, and updates. Usability problems were verified using Nielsien’s heuristics [29]. Usability tests were performed using this preliminary version of MobERAS in 10 women undergoing gynecological oncological surgery. Patients were interviewed to verify the difficulties in app use and obtain suggestions for app improvement. In this preliminary phase, system failures were analyzed and the necessary corrections and improvements were made. After the finalization of app development, comprehensive verification tests of the final product were conducted and validation tests were performed.

#### The Final Product

**Figure 3:**
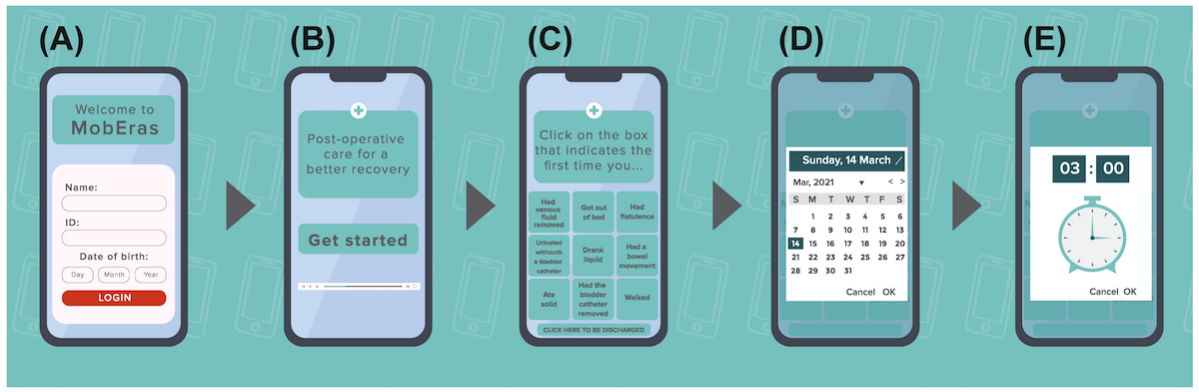
Sequence of MobERAS operation created for this study. (A) Login, (B) a video describing ERAS guidelines using drawings and visual animations, (C) the first questionnaire referring to the occurrence of events usually monitored in the postoperative period, and (D and E) the date and time of events recorded in (C). ERAS: enhanced recovery after surgery; MobERAS: mobile app for enhanced recovery after surgery.

The final product complies with General Data Protection Regulations (GDPR), guaranteeing preservation of patient privacy. The patient account registers the patient’s name, date of birth, and nationality (Figure 3A). After the patient logs in, a text with instructions on how the app works is displayed. The patient is then shown a video that uses drawings and visual animations to convey guidelines from the ERAS program aiming to improve recovery time and postoperative outcomes (Figure 3B). No intervention that causes changes in the treatment planning of the patient is proposed. The ERAS program guidelines are recommended for routine clinical and surgical practice.

**Figure 4:**
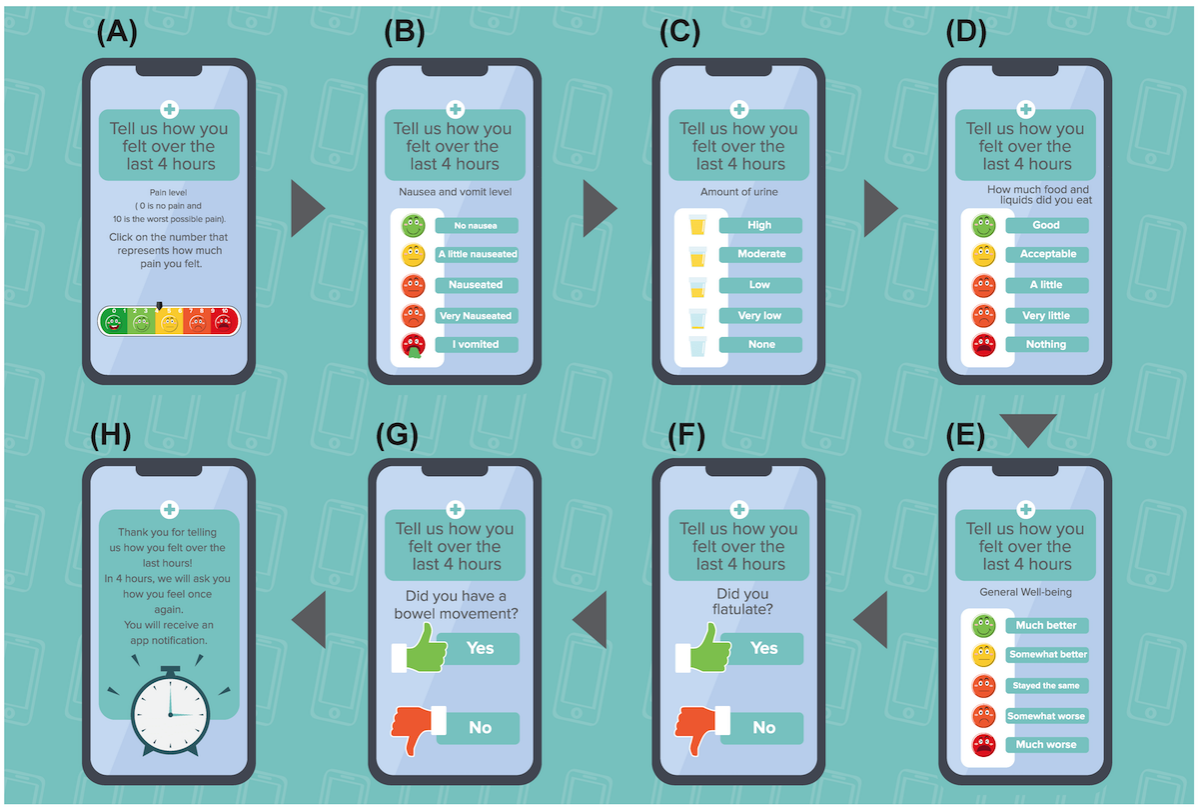
The second questionnaire, which the patient completes every 4 hours, when prompted by MobERAS. The patient is invited to click the number or figure that best represents their health status in the past 4 hours. (A) Pain level, (B) nausea and vomiting level, (C) amount of urine, (D) amount of food and liquid ingested, (E) general well-being, (F) occurrence of flatus, (G) whether bowels were opened, and (H) notice that a new notification to complete the questionnaire will be sent in 4 hours. MobERAS: mobile app for enhanced recovery after surgery.

After the end of the video, MobERAS displays a screen containing the first questionnaire, which refers to the occurrence of events usually monitored in the postoperative period, such as removal of the venous access and bladder catheter. The patient answers each question when the respective intervention occurs. The patient is also asked to record the first occurrence of certain events, such as the time of the first oral intake or spontaneous diuresis (Figure 4C-E). The events recorded by the questionnaire are presented in Textbox 1.

**Questionnaires and parameters monitored by the mobile app for enhanced recovery after surgery (MobERAS).**

**First Questionnaire**

*Click on the box that indicates the first time you*:

- Drank liquid
- Ate solid
- Had the bladder catheter removed
- Urinated without a bladder catheter
- Had flatulence
- Had a bowel movement
- Had venous fluid removed
- Got out of bed
- Walked

**Second Questionnaire**

*Tell us how you felt over the last 4 hours. Click on the figure or number that best represents your last 4 hours. *

- Visual analog scale for pain (Figure 4A): pain level (0: no pain; 10: worst-possible pain). Click on the number that represents how much pain you felt.
- Nausea and vomiting level: 5 levels of intensity (Figure 4B). No nausea, a little nauseated, nauseated, very nauseated, or vomited.
- Amount of urine: 5 levels of intensity (Figure 4C). High, moderate, low, very low, or none.
- How much food and liquids did you eat: 5 levels of intensity (Figure 4D). Good, acceptable, a little, very little, or nothing.
- General well-being: 5 intensity levels (Figure 4E). Much better, somewhat better, stayed the same, somewhat worse, or much worse.
- Did you flatulate? (Figure 4F): yes or no.
- Did you have a bowel movement? (Figure 4G): yes or no.

Simultaneously, the app screen continuously displays motivational messages on the upper scroll bar: “Try to eat all meals at the table and not in bed,” “Try to stay at least 8 hours out of bed,” “Try to walk at least 6 times throughout the day,” “Try to move your legs when you are lying down,” “Get moving! It is the best way for your recovery to be faster,” “Have you answered the questions of how you are today?” “Remember to answer the questions!”, and “Remember: your participation is essential for your recovery!”

At 4-hour intervals, alerts are triggered to complete a second questionnaire. Through this questionnaire, the patient is asked to score some scales and provide information on parameters to be monitored in the postoperative period. The patient is invited to click the number or figure that best represents their health status in the past 4 hours. These alerts are not triggered during the night (sleep period). The basic questions and scales are listed in Textbox 1, and examples of how the questions appear within the app are illustrated in Figure 4. With each positive note, the patient receives emojis or animations of recognition and congratulations to show that the event that occurred is desired for better recovery. Here, the use of gamification, through the reward system, is evident. Conversely, with each negative note, the patient receives emojis or animations to encourage improvement.

During app usage, the mobile device captures, through the GPS attached to it, the walking time and distance covered by the patient. At the time of hospital discharge, the patient confirms the date and time of the surgery and hospital discharge and then receives a final text with the guidelines to be followed at home in the postoperative period. All information recorded by the patient is sent to an online database connected to the attending physician’s cell phone, with the possibility of accessing the information in real time. This enables fast and targeted telemonitoring of patients.

### Validation of the Final Product

#### MobERAS Functioning

MobERAS was used by all 65 (100%) patients in the study. The age of the women ranged from 18 to 85 years (mean 53.2, SD 7.4) years. Regarding the level of education, 29 (44.6%) women reported a medium level, and only 2 (3.1%) said they had no education. The number of women with elementary school and higher education was similar (n=18, 28.3%, and n=16, 24%, respectively). Regarding family income, most (n=51, 78.5%) reported an income of more than 4 minimum wages, with only 1 (1.5%) woman reporting an income below 2 minimum wages.

The average time of use was 45.1 (SD 19.2) hours, ranging from 23.4 to 70 hours. All patients completed the static questionnaire. Regarding the dynamic questionnaire, a completion rate ranging from 41.7% to 100% was observed (mean 56.3%, SD 12.1%).

There was a failure to capture data related to mobility and ambulation in 5 (7.7%) patients. Of these 5 patients, 3 (60%) did not move with the mobile device, and in the other 2 (40%) cases, there was a GPS malfunction. After expert evaluation, GPS connection failure was identified. Thus, mobility and ambulation data were obtained for 60 (92.3%) of 65 patients.

#### Patients’ Evaluation of MobERAS

The average SUS score of studies available in scientific databases is 68. In this study, the patients’ perceived usability was found to be of a satisfactory level, with a mean SUS score of 81.3 (SD 9.4). Individual scores ranged from 70 to 100. The SUS score was calculated individually for each patient using MobERAS, and then, the scores were averaged. Of the 65 patients participating in the study, the majority (n=60, 92.3%) answered the final MobERAS usability evaluation questionnaire (SUS).

#### Evaluation of MobERAS by Health Care and Computer Professionals

In the evaluation of MobERAS by health care professionals and professionals specialized in technological innovation, it was possible to observe good general compliance with the proposed requirements for app evaluation. Considering a score of less than 70 as bad or very bad, from 70 to 110 as average, and higher than 110 as good or very good, the app was rated on all items evaluated according to MARS as good or very good, as shown in Table 3.

**Table 3:** MARS^a^ used in the evaluation of the quality of information contained in or provided by MobERAS^b,c^.

Dimension of MARS and assessments		Score
**Engagement**		
	Entertainment (1: dull; 2: mostly boring; 3: OK; 4: moderately fun; 5: highly fun)	137
	Interest (1: not interesting at all; 2: mostly uninteresting; 3: OK; 4: moderately interesting; 5: very interesting)	145
	Customization (1: does not allow any customization; 2: allows insufficient customization; 3: allows basic customization; 4: allows numerous options for customization; 5: allows complete customization to the individual’s characteristics)	128
	Interactivity (1: no interactive features; 2: insufficient interactivity; 3: basic interactive feat; 4: offers a variety of interactive features; 5: very high level of interactive features)	119
	Target group (1: completely confusing; 2: mostly confusing; 3: acceptable; 4: well targeted; 5: perfectly targeted)	147
**Functionality**		
	Performance (1: inaccurate response; 2: major technical problems; 3: some technical problems; 4: mostly functional with minor problems; 5: perfect response)	111
	Ease of use (1: no instructions; 2: usable after a lot of effort; 3: usable after some effort; 4: easy to learn how to use, clear instructions; 5: immediate use; intuitive)	143
	Navigation (1: disconnected and random sections/navigation difficult; 2: usable after a lot of effort; 3: usable after some effort; 4: easy to use; 5: perfectly logical and intuitive screen flow throughout)	145
	Gestural design (1: completely confusing; 2: often confusing; 3: OK, with some confusing elements; 4: intuitive, with negligible problems; 5: perfectly intuitive)	144
**Aesthetics/design**		
	Layout (1: very bad design, some options impossible to select; 2: bad design, some options difficult to select; 3: satisfactory, few problems with selecting; 4: mostly clear, able to select; 5: professional, clear device display)	118
	Graphics (1: amateurs; 2: lowqality / resolution; 3: moderate quality; 4: high quality; 5: very high quality)	122
	Visual appeal (1: no visual appeal; 2: little visual appeal; 3: some visual appeal; 4: high level of visual appeal, professionally designed; 5: very attractive, memorable, stands out)	115
**Information**		
	Accuracy of app description (1: has no description; 2: inaccurate; 3: OK; 4: accurate; 5: highly accurate description of app components/functions)	145
	Goals (N/A^d^: app goals irrelevant to research goal; 1: has no chance of achieving its stated goals; 2: has very little chance of achieving goals; 3: clear goals, which may be achievable; 4: clearly specified goals and achievable; 5: specific and measurable goals, which are highly likely to be achieved)	140
	Quality of information (N/A: no information within the app; 1: irrelevant/incoherent; 2: barely relevant/coherent; 3: moderately relevant/coherent; 4: relevant/coherent; 5: highly relevant/coherent)	144
	Quantity of information (N/A: no information within the app; 1: minimal; 2: insufficient; 3: OK but not comprehensive; 4: broad range of information, some gaps; 5: comprehensive and concise, contains links to more information and resources)	117
	Visual information (N/A: no visual information within the app; 1: completely confusing; 2: mostly confusing; 3: OK but often confusing; 4: mostly clear; 5: perfectly clear)	147
	Credibility (1: legitimacy worthiness of source questionable; 2: legitimate source but cannot be verified; 3: developed by small nongovernmental organization/institution/specialised commercial business/funding body; 4: developed by government, university, or as above but larger in scale; 5: developed using nationally competitive government or research funding)	120
	Evidence base (N/A: app not tested; 1: app not working; 2: trialled and partially positive outcomes in studies that are not RCTs^e^; 3: trialled and positive outcomes in studies that are not RCTs; 4: trialled and positive outcomes in 1-2 RCTs; 5: trialled and positive outcomes in >3 high-quality RCTs)	N/A

^a^MARS: Mobile App Rating Scale.

^b^MobERAS: mobile app for enhanced recovery after surgery.

^c^Adapted from Stoyanov et al [28].

^d^N/A: not applicable.

^e^RCT: randomized controlled trial.

## Discussion

### Principal Findings

This study described the development and validation process of an app (MobERAS) for telemonitoring, guiding, and encouraging the adoption of medical recommendations for use in the postoperative period. To the best of our knowledge, this is the first study to describe such an app for telemonitoring patients in the postoperative period using gamification. There are few apps developed to be used in the perioperative period, and most of these apps are intended to provide information to patients. An example is the Heal Better app [30], which aims to help educate and empower patients to comply with a recovery care plan after abdominal surgery by providing clinical information. The difference between MobERAS and these other apps is the real-time monitoring of the patient in the postoperative period and the use of gamification as a tool to encourage recommended medical practices. By combining telemonitoring with gamification, MobERAS aims to (1) monitor the postoperative course using data provided by patients; (2) evaluate the incorporation of the ERAS program recommendations into clinical and surgical practice and patients’ responses to the adopted recommendations; (3) promote greater participation of patients in their postoperative recovery process and better adherence to medical recommendations, aiming to achieve faster recovery, early discharge from the hospital, and lower rates of complications; and (4) enable remote and real-time access of health data and postoperative evolution by physicians.

Worldwide, over 300,000 health apps are currently under development. Both the US Food and Drug Administration (FDA) and the European Medicine Agency of the European Union (EU) have recognized the importance of the software in diagnostic and therapeutic devices. Guidelines related to the use of digital tools in health were published by the World Health Organization in 2018, organizing the different digital and mobile interventions and their use in favor of the health system [31]. Significant progress has been made in app development, building an evidence base, validating functionality, and creating standards for development and design structures for app reviews. Nonetheless, even if an app is well developed, with evident quality, its ability to improve the health and well-being of patients can only be achieved if the app is actually used [1]. Features such as gamification can significantly increase users’ attention and involvement. The most accepted definition of gamification is “the use of game design elements in nongame contexts.” There are many gamification strategies that increase engagement, such as narrative structure, symbols, or avatars based on self-image, as well as integration tutorials. Resources present in smartphones, such as sensors and GPS services, have been useful for gamified health care interventions [15]. Considering this, gamification strategies were used to develop MobERAS, including the use of figures representing the health status of patients, a reward system, avatars, and motivational figures, as well as a GPS attached to the system to monitor walking. We believe that this will motivate patients to fulfill their goals and consequently follow medical recommendations, thus reaching the desired postoperative objectives.

It is known that walking 10 m (or 30 steps) without interruption can reduce the risk of thromboembolic phenomena by up to 50% [32]. It is also known that mobile device tools, such as sensors, can be useful in the delivery of health care resources [28]. Mobile devices have sensors integrated into them, and this type of resource can assist health care professionals in treating their patients with permanent connectivity [33]. In this context, the GPS integrated into MobERAS is used for continuous monitoring of mobility time and walking distance.

MobERAS was designed to start being used in the postoperative period for patients still in the hospital, showing the first signs of occurrence of important events, such as bladder catheter removal or the first oral intake, in addition to collecting postoperative data at short intervals, considering the need for more frequent monitoring during the hospital stay, such as monitoring pain, diet acceptance, or diuresis volume. For this reason, the app was validated in the hospital in the postoperative period within less than 72 hours of use. Nonetheless, the app can be programmed to collect information at longer intervals (eg, once or twice a day), and its domestic use can be evaluated in another study.

MobERAS collects self-reported data from patients just as if they were having an appointment with a doctor. The idea is for the app to become a tool to help with postoperative monitoring and not a replacement for regular supervision by the health care team. All information received by health care professionals in real time enables the early diagnosis of possible postoperative complications, resulting in better patient supervision after surgery being able to prevent more serious complications.

Postoperative adverse events are associated with longer hospital stays and increased mortality rates. The ERAS program represents a paradigm shift in conventional perioperative care, replacing some traditional practices with better evidence-based practices, minimizing overall health care spending, allowing for faster and safer rehabilitation, and improving well-being and patient satisfaction [15,16]. With this objective, the program focuses mainly on a decrease in perioperative stress, adequate pain control, return to normal gastrointestinal function, and early mobilization [22]. The app developed and described in this study was based on recommendations of the ERAS program. The parameters evaluated are those that can influence the length of hospital stay, such as nausea, vomiting, pain, return to normal normal gastrointestinal function, and thromboembolic events.

Thus, according to the guidelines recommended by the ERAS program and because MobERAS is gamified, we believe that the app can encourage longer walking times, with a consequent faster return to normal gastrointestinal function, and fewer thromboembolic events, resulting in benefits such as faster postoperative recovery, shorter hospital stay, and a lower rate of postoperative complications. Through the continuous collection of data from patients and the sharing of this knowledge with the doctor in real time, better pain control and better management of nausea and vomiting are expected with the use of the app. Moreover, the hospital will benefit from lower hospitalization costs without increasing the rate of complications.

Although mobile device ownership is widespread, its use in disease management and self-care is still in its early stages, and there is limited knowledge about its use in the perioperative setting [12]. MobERAS has as some of its objectives the collection of postoperative data and the telemonitoring of patients undergoing surgery. Using validation tests, these objectives were achieved, with the collection of clinical data and the monitoring of patients’ health status, sharing information with health care professionals in real time. Objective symptoms are traditionally monitored as part of care and treatment, but patients’ subjective descriptions are considered a key element of monitoring [15]. Through the app, patient self-reported information was collected, both regarding symptoms, such as pain, nausea, and vomiting, and objective parameters, such as serum and catheter withdrawal times.

Most health apps for smartphones have simple functions and do a little more than providing basic information. However, there is great potential for developing more effective gamified apps, depending on the repertoire and combinations of techniques used that are appropriate for a gamified platform. This development requires multidisciplinary collaboration between game developers, behavior change specialists, and public health experts [34]. In response to this need, this study involved health care professionals and experts in innovation technology in both the development and evaluation of MobERAS. As a result, we developed an app that, in addition to collecting data, uses gamification as a tool to guide and encourage patients to engage in their postoperative recovery process and comply with medical recommendations.

Given the rapid proliferation of smartphone apps, it is increasingly difficult for users, health care professionals, and researchers to identify and evaluate high-quality apps. Little information about the quality of apps is available [29]. One of the critical issues is the lack of evaluation of the reliability of mHealth monitoring systems [33]. In addition, there is little evidence that health care professionals and users participate in the design of health apps, and most apps do not contain theoretically consistent behavior change techniques. Few apps are compliant with the regulatory processes or have had their effectiveness formally evaluated, leading to concerns about the lack of benefits or even potentially harmful apps [34]. For this reason, we chose to perform a comprehensive validation of our app in this study, with quality assessment both from a subjective point of view, using the SUS answered by patients using the app, and from a technical point of view, using MARS and expert assessments.

Through the SUS answered by the patients, good evaluation was observed regarding usability, which suggests good acceptance of the use of the app by the patients. The SUS was chosen because of its widely recognized use in assessing product usability and its flexibility to adapt, because it is quick and easy to use, and because it offers a unique score that is easily understood by people who are involved in product and service development [26]. In addition, MobERAS was rated by the experts as good or very good on all MARS parameters, which also suggests a well-developed app with regard to engagement, functionality, design, and information. This leads us to conclude that this is a safe app with potential benefits and possible applicability in clinical practice.

### Limitations and Strengths

One limitation of this work is that no case-control study was conducted to assess the impact of using the software on the occurrence of postoperative complications and the length of hospital stay. Furthermore, the use of MobERAS depends on an internet connection and active patient participation, and there may be changes in the level of awareness, pain, and other parameters that limit the correct use of MobERAS during the early postoperative period. The gamification process and app design also require further improvement. Although the app involved participation of patients during initial construction of the product and validation tests were performed with the participation of patients and professionals, the app was not codesigned with patients and health care professionals. In addition, the difficulty or ease of use of the app according to different age groups and socioeconomic levels was not analyzed.

However, the study also highlights the strengths of the app and its usefulness, which include the contribution of a multidisciplinary team and international applicability. Moreover, the technology used is compatible with current trends in health practices, and the app is compatible with cell phones and devices present in the daily life of the population. Another important point is that MobERAS was examined by experts, with good evaluation. In addition, the app’s good functionality was proven, with postoperative data capture and its potential clinical applicability, in addition to good acceptance of its use by the patients.

### Challenges

Challenges in the field of digital health care include issues related to development, validation, and use [35]. Despite such challenges, it is expected that these obstacles will be overcome by a growing number of apps that will become increasingly suitable for use in clinical practice. However, the first challenge in the dissemination of apps for clinical use is to increase awareness of the technologies available to doctors [1]. We believe that MobERAS can be of great help in the health care of patients, with possible early detection of postoperative complications, in addition to better monitoring of patients. We believe that the app can contribute to the health team acting immediately and more effectively when facing complications. Moreover, because the app is easy to navigate and attractive through the use of gamification tools, the patient can be stimulated to follow medical recommendations for better recovery. The app will work as a “virtual companion” during the patient’s postoperative stay in the hospital. A subsequent randomized clinical trial will help us understand better the impact of MobERAS on postoperative clinical practice.

### Conclusion

In conclusion, MobERAS can be used to orient patients, obtain postoperative data, and monitor patients in real time. The app is easy to use and attractive, given its gamified design. MobERAS is well accepted by patients and well evaluated by experts. Overall, MobERAS can be of great use in clinical practice, promoting the engagement and commitment of patients in their postoperative care. We believe that the app can contribut to better postoperative assistance and outcomes. Further studies are required to verify the clinical applicability of MobERAS in a greater number of patients and the impact of its use on postoperative recovery.

## Abbreviations

ERAS: enhanced recovery after surgery
MARS: Mobile App Rating Scale
mHealth: mobile health
MobERAS: mobile app for enhanced recovery after surgery
RCT: randomized controlled trial
SUS: System Usability Scale
